# Bambara Groundnut Rhizobacteria Antimicrobial and Biofertilization Potential

**DOI:** 10.3389/fpls.2022.854937

**Published:** 2022-07-13

**Authors:** Caroline F. Ajilogba, Olubukola O. Babalola, Patrick Adebola, Rasheed Adeleke

**Affiliations:** ^1^Niche Area for Food Security and Safety, Faculty of Natural and Agricultural Science, North-West University, Mmabatho, South Africa; ^2^Agricultural Research Council-Natural Resources and Engineering, Pretoria, South Africa; ^3^Agricultural Research Council-Vegetable and Ornamental Plants, Pretoria, South Africa; ^4^International Institute of Tropical Agriculture, Abuja, Nigeria; ^5^Unit for Environmental Sciences and Management, North-West University, Potchefstroom, South Africa

**Keywords:** bambara groundnut, biocontrol, biofertilizer, plant growth-promoting rhizobacteria, rhizosphere, *Bacillus*, rhizobacteria

## Abstract

Bambara groundnut, an underutilized crop has been proved to be an indigenous crop in Africa with the potential for food security. The rhizosphere of Bambara groundnut contains Rhizobacteria, with the ability to grow, adapt, and colonize their surroundings even in unfavorable conditions and have not been explored for their plant growth-promoting properties. The aim of this research was to determine the potential of rhizobacteria from Bambara groundnut soil samples as either biofertilizers or biocontrol agents or both to help provide sustainable agriculture in Africa and globally. Bambara groundnut rhizospheric soil samples were collected and analyzed for their chemical composition. Rhizobacteria isolates were cultured from the soil samples. Plant growth-promoting, antifungal activities and phylogenetic analysis using 16S rRNA were carried out on the isolates to identify the rhizobacteria. A 2-year field study planting was carried out to determine the effect of these rhizobacteria as biofertilizers for Bambara groundnut (*Vigna subterranean*). The study was carried out in a complete randomized block experimental design with three replications. All the isolates were able to produce ammonia and 1-aminocyclopropane-1-carboxylate, while 4.65, 12.28, and 27.91% produced hydrogen cyanide, indole acetic acid, and solubilized phosphate, respectively, making them important targets as biocontrol and biofertilizer agents. The field results revealed that treatment with rhizobacteria had significant results compared with the control. Characterization of selected isolates reveals their identity as *B. amyloliquefaciens, B. thuringiensis*, and *Bacillus* sp. These Bacillus isolates have proved to be plant growth-promoting agents that can be used as biofertilizers to enhance the growth of crops and consequent improved yield. This is the first time the rhizobacteria from the Bambara groundnut rhizosphere are applied as biofertilizer.

## Introduction

The use of chemicals to inhibit the growth of pathogenic microorganisms in plant disease control has been a global issue. Research for healthier environmental control methods has led to biocontrol and biofertilization. Rhizospheric soils of legume crops have been considered a reservoir for plant growth-promoting rhizobacteria (PGPR). Bambara groundnut (*Vigna subterranean* L. Verdc), a legume crop, is one of the neglected and underutilized crop species (NUCS). The term ‘NUCS’ is used to mean wild species of plant, which are non-commodity cultivated. They form part of a large agro biodiversity portfolio that is not used as a result of an array of factors, such as agronomic, genetic, economic, social, and cultural factors (Chivenge et al., 2015; Mudau et al., 2022). They are traditionally grown by subsistence farmers in their various localities where they are useful in supporting and securing nutrition in local communities in order to meet their sociocultural needs and traditional uses. They have been largely ignored by research and development and so there is no competition for them compared to other well-established major crops. This results in the loss of both their diversity and traditional knowledge. It is a food known as a balanced diet as it contains the right proportion of protein (16.25%), carbohydrate (∼64.4%), fats (6.3%), fiber (5.5%), and is rich in minerals (Tan et al., 2020). The protein is high in both lysine (6.6%) and methionine (1.3%) (Ajilogba et al., 2022a). Because of its richness in protein and the fact that it is nutritious, it is a source of food security, especially for small-scale farmers and small households (Tan et al., 2020). Bambara groundnut is also very rich in micronutrients, such as potassium, calcium, and iron, with a high proportion of fiber (Mubaiwa et al., 2017). There are different varieties with varying mineral composition, for example, the red varieties contain iron two times as much as the cream variety, making it quite suitable for mineral deficient in iron (Mubaiwa et al., 2018). It was observed that fermentation of bambara groundnut helped to improve its mineral composition, which invariably reduced the different factors that inhibited nutrient utilization, such as trypsin, oxalate, and phytic and tannic acid (Olanipekun et al., 2015).

Bambara groundnut has the ability to grow under different climatic and soil conditions that are harsh and extreme, thus making it suitable to be grown in semiarid lands. The soil rhizosphere of legumes, which include bambara groundnut, has been indicated to enhance plant growth and also for controlling plant pests and diseases (Ajilogba and Babalola, 2019; Ajilogba et al., 2022a). Such beneficial attributes are associated with a host of rhizobacteria that inhabit this rhizosphere and are also sometimes referred to as plant growth-promoting rhizobacteria (PGPR). Examples of such bacteria include *Bacillus* spp, *Actinomycetes*, spp *Pseudomonas* spp, *Burkholderia* spp, and *Rhizobium* spp (Ajilogba and Babalola, 2013, 2016; Ajilogba et al., 2013). The diversity of these microbial communities is driven by plant-microbe activities, such as an organic compound secreted by plants, as well as availability and quantity of nutrients released by microbes (Ajilogba and Walker, 2020). PGPR also promote plant growth directly by producing phytohormones, such as Indole Acetic Acid (IAA), and indirectly by producing HCN, which is toxic against plant pathogen (biocontrol) and also makes phosphate available to plants (Ajilogba and Babalola, 2019; dos Santos et al., 2020; Ajilogba et al., 2021; Kumar et al., 2021). Sometimes, they enhance iron chelation (siderophore production) and supply of nutrients, such as phosphorus (phosphate solubilization) and nitrogen (nitrogen fixation), to also promote plant growth. They are well-known to participate in biofertilization, which involves enriching rhizospheric soil, making nutrients available to the plants, as well as aiding the plants in nutrient uptake and the subsequent use of the nutrients for metabolic processes by the plants (dos Santos et al., 2020; Kumar et al., 2021). They have also been found to help in biocontrol of plant pests and diseases by suppressing and/or inhibiting the growth of pathogens in/on plants (Ajilogba et al., 2017; Kumar et al., 2021). As new pathogens causing plant diseases are being identified, there is the need to find better bio-alternatives that have not been harnessed but have prospects. The rhizosphere of bambara groundnut has not been explored like other legumes for rhizobacteria that are important in biofertilization and biocontrol. This study aimed at evaluating the rhizobacteria found in the rhizosphere of Bambara groundnut for their biofertilization and biocontrol potentials as a tool for food security. This has been, so far, the first field study on biofertilizer effect of PGPR on the growth of bambara groundnut.

## Materials and Methods

### Planting of Bambara Groundnut

**FIGURE 1:**
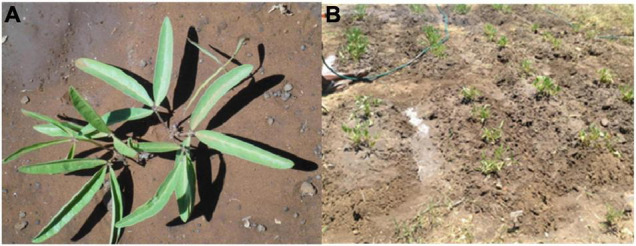
A plot showing Bambara groundnut 2 weeks after planting **(A,B)**.

The propagation of bambara groundnut was through seeds on level seedbeds or ridges where the soil is wet. Bambara groundnut seeds were planted on seedbeds in plots that were 50 cm apart, and spacing between seed holes on each plot was 50 cm apart. Bambara groundnut seeds were planted 3–4 cm deep in the soil (2–3 seeds per hole). Twenty-five (25) plots were cultivated for each replication, and the experiment was repeated three times for the first planting season (2014/2015), which was also used as control (Figures 1A,B).

### Soil Sampling and Collection

Soil samples were collected from field trials during the planting period between October 2014 and March 2016 from the North-West University Agricultural Farm, Mafikeng Campus (Lat., 25°78′91″ Long., 25°61′84″) Mafikeng, South Africa according to Ajilogba and Babalola (2019) and Ajilogba et al. (2021, 2022b).

### Soil Analysis

According to the International Standard Organization (ISO) standard 11464, the samples were prepared for analysis by drying at room temperature, pulverized, and sieved through a 2-mm sieve. All glassware used for soil analyses was washed thoroughly, soaked in 20% nitric acid, and rinsed with deionized water to prevent the presence of impurities. The selected physical and chemical parameters of the samples were analyzed using standard laboratory procedures (Gavrićet al., 2019). All soil analysis was repeated two times.

#### Physical and Chemical Analysis of Samples

##### Determination of Cation Exchange Capacity and Extractable Cations

Exchangeable cation was determined using the ammonium acetate method according to Gumbara et al. (2019) with slight modification. The adsorbed NH_4_ was determined by the aeration method.

##### Nitrogen Analysis of Soil Samples

Soil samples were dried at 80°C, grounded to a powder, and 1 g analyzed for nitrogen (N) by Kjeldahl digestion (Ameen et al., 2019).

##### Determination of Nitrate Composition

Nitrate contents of the samples were determined using the equilibrium extraction method. Ten (10) g ≤ 2. mm of air-dry soil was placed into a 250-ml wide mouth extraction bottle, and 100 ml of 0.1 mol dm^–3^ of KCl was added, stoppered, and shaken for 30 min on a shaker. The solution obtained was filtered to get a clear extract, and the nitrate contents were determined in the clear extract.

##### Determination of pH and Redox Potential

Ten (10) g of soil was weighed and mixed with 25 ml of distilled water to obtain the ratio 1:2.5 (m:v) soil-water suspension and left to shake for 1 h and left standing overnight for pH measurement, with a pH meter Jenway 3520™ (Lasec, South Africa). The pH meter was standardized using calibration buffers 4, 7, and 9. The combined electrode was inserted into supernatant, pH values and redox potential of the samples were recorded, and electrode was washed with distilled water after each reading.

##### Determination of Organic Matter

Organic carbon was determined by the Walkley-Black method (Bierer et al., 2020). The percentage of organic matter was used to calculate the organic carbon content by using the conversion factor 1.724 and the fact that 58% of the soil organic matter is the average content of carbon. This calculation is given below:

Percentage organic matter in soil is calculated as written below:


$$\mathrm{Molarity}\left(M\right)=\frac{10}{\mathrm{vblank}}=\frac{10}{9.6}=1.041$$

$$\%\mathrm{oxidizable}\mathrm{organic}\mathrm{carbon}=\frac{\left[\mathrm{vblank}-\mathrm{vsample}\right]x0.3xM}{\mathrm{Wt}}$$

(wt = weight of air-dried soil = 1 g)

% total organic carbon (w/w) = 1.334 x % oxidizable organic carbon

% organic matter (w/w) = 1.724 x % total organic carbon

### Preparation of Soil Samples for Bacterial Isolation

Soil samples from the rhizosphere of Bambara groundnut and preparation for bacterial isolation were carried out according to Ajilogba and Babalola (2019).

### Culturing and Isolation of Bacteria From Soil Samples

Isolation and enumeration of bacteria present in the soil sample were performed by the serial dilution plate technique using tryptone soy agar (TSA) according to Ajilogba and Babalola (2019). Viability was confirmed by the standard plate count method using tryptone soy broth plus 2% agar (TSBA). These inocula were prepared to use them *in vitro* for testing the antifungal and biocontrol activities of the isolates (Cavaglieri et al., 2005).

### Plant Growth-Promoting Rhizobacteria and Biochemical Analysis of Bacteria Isolates

#### Detection of Hydrogen Cyanide Production

Bacterial isolates were screened for the production of hydrogen cyanide (HCN) production according to the methodology previously described by Lorck (2006) and Rijavec and Lapanje (2016), with slight modifications. Bacterial cultures were streaked on a nutrient agar medium containing 4.4 g per liter of glycine. Picric acid solution (0.5% in 2% sodium carbonate) was prepared, and Whatman filter paper No. 1 was soaked in it and was placed inside the lid of a plate, which was sealed with parafilm. After plates were incubated at 30°C for 4 days, production of HCN was observed by the light brown to dark brown color that developed, and no coloration development indicated negative activity.

#### Determination of Indole Acetic Acid Production

Fifty (50) ml of nutrient broth (Merck) containing 0.1% (D) L-tryptophan was inoculated with 500 μL of 24-h old bacterial cultures and incubated in a refrigerated incubator Shaker at 30°C and 180 rpm for 48 h in the dark. The bacterial cultures were then centrifuged at 10,000 rpm for 10 min at 4°C, and detection of the presence of indole acetic acetic was carried out according to Gang et al. (2019).

#### Determination of Phosphate Solubilization

Bacterial isolates were spot inoculated on Pikovskaya agar medium plates. The plates were incubated at 28°C for 7 days. Phosphate solubilization (PS) activity was observed as a clear zone around the colonies, while no zone was considered negative activity (Rai et al., 2018; Suleman et al., 2018).

#### Detection of Ammonia (NH_3_) Production

Peptone water was used to determine ammonia production of bacterial cultures. Freshly grown cultures were inoculated into 10-ml peptone water and incubated for 48–72 h at 30°C. Nessler’s reagent (0.5 ml) was added in each tube after incubation, and a positive test was observed as brown to yellow color development, while negative activity was observed with no color development (Karthika et al., 2020).

#### Determination of 1-Aminocyclopropane-1-Carboxylate

This procedure was carried out according to the protocol of Li et al. (2011). Ninhydrin reagents were prepared, and five working concentrations of 1-Aminocyclopropane-1-Carboxylate (ACC) were used, which were 0.05, 0.15, 0.2,0.3, and 0.5 mmol^–1^ for a colorimetric assay using the 96-well PCR plates. Absorbance was read at 570 nm.

#### Catalase Activity

A sterile toothpick was used to mix 48-h-old bacterial colonies placed on a clean glass slide to which a drop of 3% hydrogen peroxide was added. The effervescence that follows indicated catalase-positive activity, while no effervescence indicated negative activity.

#### Assay for Protease Production

Extracellular protease production was assayed according to Laili and Antonius (2017), with slight modifications. Spot inoculation of each bacterial isolate on a skim milk agar plate was carried out and incubated at 37°C for 24 h. Development of the halo zone around the bacterial colony was considered as a positive test for protease production, while absence of the halo zone was considered as a negative test.

#### Oxidase Activity

Oxidase activity was determined by using the modified filter paper spot method according to Shields and Cathcart (2010). Kovács oxidase reagent (1–2 drops) was added to 24-h-old culture on a small piece of filter paper. Change in color to dark purple within 60–90 s was considered as the oxidase positive test, while absence of color change indicated negative activity. All analyses were repeated two times.

### Antifungal Effect Assay

Antifungal activities of isolates against *F. graminearum*, which is toxin-producing fungi and pathogenic to man, animals, and plants, were carried out according to Ajilogba and Babalola (2019).

### Antibacterial Effect Assay

Antagonistic activity of isolates against *B. cereus* and *E. feacalis* was carried out according to Ajilogba and Babalola (2019).

### Metabolites Detection

Metabolites characterization from antifungal and antibacterial assays was carried out as reported by Ajilogba and Babalola (2019).

### Isolation of Genomic DNA

Genomic DNA of all isolates was extracted using a ZR soil Microbe DNA MiniPrep™ (Zymo Research, Irvine, CA, United States) extraction kit. This was carried out according to the manufacturer’s manual (Ajilogba and Babalola, 2013).

### PCR Amplification Targeting the 16S rRNA

Molecular identification of isolates was done by PCR amplification of 16S rRNA using the universal bacterial primers F1 (5′-GAGTTTGATCCTGGCTCAG-3′) and R2 (5′-GWATTA CCGCGGCKGCTG-3′) according to Ajilogba and Babalola (2019) for isolates BAMji, BAMr, BAMui, BAMx, BAMuii, and BAMxi. A 1.5-kb fragment was amplified from the genomic DNA of isolates BAMbi, BAMhi, BAMli, BAMpii, BAMrii, and BAMxii, with the bacterial universal primers 27F (5′-AGAGTTTGATCMTGGCTCAG-3′) and 1492R (5′CGGTTAC CTTGTTACGACTT-3′).

### Sequencing and Phylogenetic Analysis

The sequencing of the purified PCR products was conducted according to Ajilogba and Babalola (2019). Phylogenetic analyses were conducted using softwares in MEGA version 5.2.2. Evolutionary distance matrices were generated, and a phylogenetic tree was inferred by the neighbor-joining method (O’Sullivan et al., 2021). Tree topologies were evaluated by bootstrap analysis (Tekdal et al., 2022) based on 1,000 resamplings of the neighbor-joining data set. Manipulation and tree editing were carried out using TreeView (Soh et al., 2012).

### Experimental Design

This experimental design was a completely randomized design (CRD) with four treatments replicated three times. The treatments were with identified isolates *B. amyloliquefaciens, B. thuringiensis, Bacillus* sp., and the control.

### Preparation of Rhizobacteria From Bambara Groundnut Rhizosphere for Seed Inoculation

#### Preparation of Inoculum

Preparation of rhizobacteria inoculum was carried out according to El-Shabrawy and Shehata (2018). Two loopfuls of each of the bacteria from 3-day-old cultures on tryptic soy agar (TSA) were transferred separately to a 50-ml tryptic soy broth (TSB) medium and incubated overnight at 28 ± 2°C. Viability was confirmed by the standard plate count method using tryptone soy broth plus 2% agar (TSBA) (Ajilogba et al., 2013). The inocula for use in the field were prepared according to Ajilogba et al. (2013).

#### Bambara Groundnut Seed Propagation for Plant Growth-Promoting Rhizobacteria

By the second planting season, the second year, 2015/2016, treatments with *B. amyloliquefaciens, B. thuringiensis*, and *Bacillus* sp. were applied to bambara groundnut seeds before planting on a freshly cultivated soil. Samples were taken from different ridges or seedbeds, each having three rows per ridge, each row having five seed holes, and having three seeds per seed hole, making a total of 15 seeds by row and 45 seeds by ridge or 45 plants per ridge.

#### Determination of Growth Parameters

The following growth parameters were analyzed in the field; they are leaf number, length of leaf, breadth of leaf, number of stems, length of stems, roots, and shoot length and number of seeds. At the end of the developmental stages [BGN vegetative (4 WAP), flowering (8 WAP), pod-filling (12 WAP), and the maturation stage (16 WAP)], one plant from each planting hole was carefully scooped with all its roots intact using a trowel and hand washed over a fine sieve with tap water, collecting all roots one at a time. A meter rule was used to measure plant growth parameters, while the seeds were counted.

### Data Analysis

On growth parameters, ANOVA was performed for comparison of means. Significant means was separated using the Tukey–Kramer honest significant difference (HSD) test at the 5% level, and correlation analysis was performed to determine the relationship between variables at the 5% level. On the bacterial isolates, phylogenetic tree construction was carried out from the sequences of DNA, and the generational distances between isolates were calculated.

## Results

### Physical and Chemical Characterization of Samples

#### Cation Exchange Capacity, Nitrate, and Nitrogen Analysis of Soil Samples

**FIGURE 2:**
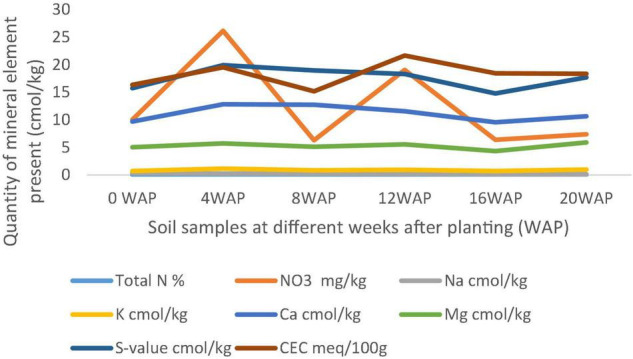
Comparison of different physical and chemical properties of soil samples between 4 and 20 weeks after planting (WAP**).** N, nitrogen; K, potassium; Ca, calcium; Na, sodium; Mg, magnesium; S-value shows the mean of the whole graph.

In Figure 2, it was observed that the exchangeable cations and the nitrate value followed the same trend in the graph. They decreased from the 4 WAP to the 8 WAP and increased again at the 12 WAP, which was the peak, and decreased again at the 16 WAP only to start increasing gradually to the time of harvest. The nitrate content ranged between 6.28 mg/kg at the 8 WAP and 26.16 mg/kg at the 4 WAP. From the 12 WAP, it decreased again from 19.06 mg/kg to 6.40 mg/kg at the 16 WAP and gradually increased again to the time of the harvest to 7.39 mg/kg.

**FIGURE 3:**
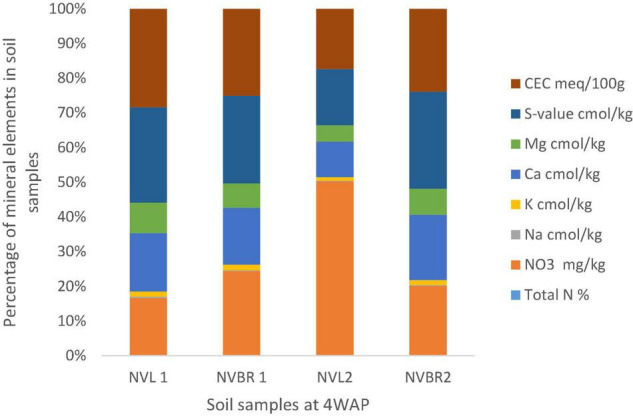
Physical and chemical analysis of soil samples at 4 WAP. Nitrate (NO3) and CEC were the highest quantity of mineral found in the soil samples, and both were found in NVL2 and NVL1, respectively. N, November; VL and VBR, variety of Bambara from whose root, soil samples (NVL1, NVL2, NVBR1, NVBR2) were taken.

**FIGURE 4:**
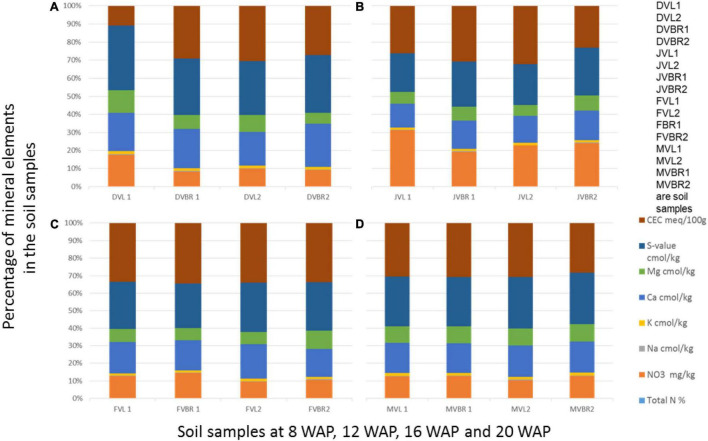
Physical and chemical analysis of soil samples at 8, 12, 16, and 20 WAP. DVL1, DVL2, DVBR1, and DVBR2 are soil samples from 8 WAP **(A)**, JVL1, JVL2, JVBR1, and JVBR2 are soil samples from 12 WAP **(B)**, FVL1, FVL2, FVBR1, and FVBR2 are soil samples from 16 WAP **(C)**, MVL1, MVL2, MVBR1, and MVBR2 are soil samples from 20 WAP **(D)**, D, December; J, January; F, February; M, March; VL and VBR, variety of Bambara from whose root soil samples were taken.

All chemical parameters, N, K, Ca, Na, and Mg increased from zero in the original bulk soil to 4 WAP. Cation exchangeable capacity (CEC) and nitrate followed the same pattern of decreasing from the time of fertilization/flowering (4 WAP) till the 8 WAP and increasing at 12 WAP. They gradually decreased from 12 WAP to 16 WAP and gradually increased again to the time of harvest. Samples at 12 WAP had the highest CEC of 21.64 meq/100 g. The lowest was 15.19 meq/100 g at 8 WAP. Nitrate (NO_3_) was highest at 4 WAP and lowest at 8 WAP. After 4 WAP, all the minerals kept decreasing gradually until the 16 WAP (which is the time of maturity of seeds) and then started increasing gradually again. The only exceptions are the individual cations Mg, Na, K, and Ca, which all followed the same pattern of gradually decreasing up until the 16*^th^* week and then gradually increasing afterward to the time of the harvest (Figures 3, 4).

Furthermore, Figure 4A revealed that CEC and Ca were the highest mineral found in the soil samples, and both were found in DVL2 and DVBR2, while NO3 decreased and total N was seen to increase from the previous weeks where it was absent in all the samples. Figure 4B showed that CEC and NO3 were the highest mineral found in the soil samples, and both were found in JVL1 and JVL2; Figure 4C showed that CEC is highest and found in FVBR1, while NO_3_ has decreased again but available in all the samples. Figure 4D showed that CEC is highest and found in MVBR1, while NO_3_ has gradually increased again and available in all the samples. Also, total N increased and consistent in Figures 4B–D samples.

#### pH and Redox Tolerance of Soil Samples

**FIGURE 5:**
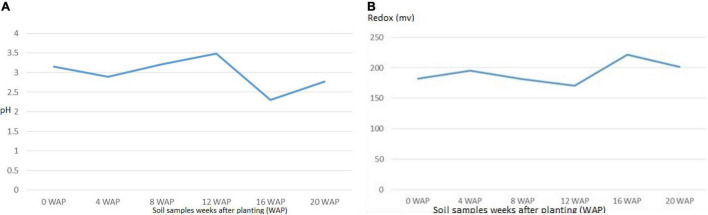
**(A)** Average pH values of soil samples from original soil to the time harvest. The highest pH was at 12 WAP, while the least was at 16 WAP. **(B)** Average Redox value for soil samples at the different growth stages from original soil to the time harvest. The highest value was at 16 WAP, while the lowest was at 12 WAP. The line graph for redox and pH is inversely related.

The different soils at the different growth stages differed in their physical and chemical properties. The pH reduced gradually from the original bulk soil to the soil at the time of harvest. The pH ranged from 2.3 at 16 WAP, which was the lowest, to 3.4 at 12 WAP, which was the highest. The reduction-oxidation relationship of both living and non-living things is measured by the redox potential (Eh) measured in volts. The Eh in this study ranged from 170.33 mV at 12 WAP to 221.67 mV at 16 WAP. This pattern of Eh and pH shows that they are both negatively correlated. This result reveals that nitrogen fixation by root nodule bacteria and activities of rhizospheric bacteria can affect the condition of the soil from one season to the other (Figures 5A,B). Also, although redox value, <300 mV can be limiting for plant growth, but bambara groundnut has grown and also increased in yield.

### Organic Matter Content of Soil Samples

**FIGURE 6:**
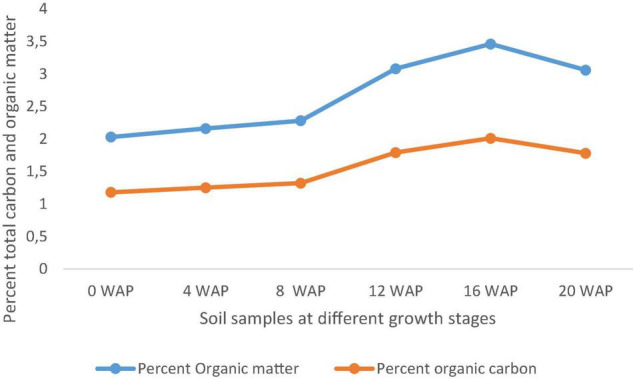
Carbon and organic matter content of soil samples from 0 WAP to harvest (20 WAP).

The organic matter of soils varies based on the type of soil. The organic matter in this study ranged from 2.02% at 0 WAP (bulk soil), which is the lowest to 3.46%, which is the highest at 16 WAP. The organic matter kept increasing form 4 WAP to 16 WAP and reduced at the time of harvest. Total organic carbon also followed the same pattern as the total organic matter. It ranged from 1.18% at 0 WAP, which was the lowest to 20.01% at 16 WAP, which was the highest (Figure 6).

### Culturing and Isolation of Bacteria From Soil Samples

The 43 isolates subcultured spanned through the different growing seasons. Some isolates that grew at the beginning of the growth period were also isolated at harvest, while most of them were not. Most of the organisms isolated at the time of the harvest were not the same as those that were there from the beginning. The number of isolates increased from 4 WAP to 8 WAP only to decrease at 12 WAP and then continued increasing up till the time of the harvest. The period of the 12 WAP corresponded to the pod and seed formation period (Table 1).

**TABLE 1:** Total number of different isolates by morphology at different growth stages.

Soil samples (4 WAP)	*NOI	Soil samples (8 WAP)	*NOI	Soil samples (12 WAP)	*NOI	Soil samples (16 WAP)	*NOI	Soil samples (20 WAP/Harvest)	*NOI
NVL 1	3	DVL 1	5	JVL 1	3	FVL 1	3	MVL 1	22
NVBR 1	2	DVBR 1	9	JVBR 1	2	FVBR 1	4	MVBR 1	17
NVL2	8	DVL2	8	JVL2	5	FVL2	11	MVL2	8
NVBR2	–	DVBR2	2	JVBR2	3	FVBR2	4	MVBR2	–
Total	**13**		**24**		**13**		**22**		**47**

*NOI is number of isolates. Values in bold are the total number of rhizobacteria isolated at each growth stage.

### Plant Growth-Promoting Rhizobacteria Activities

**FIGURE 7:**
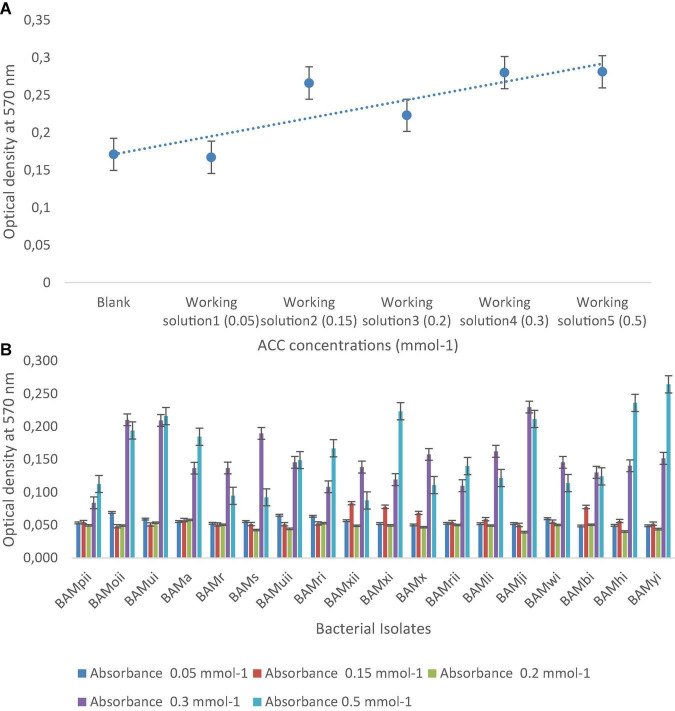
**(A)** The standard curve of ACC concentrations, ranging from 0.05 to 0.5 mmol^– 1^ determined by the 96-well PCR-plate ninhydrin assay (*y* = 0.0242x + 0.1467, *R*^2^ = 0.7364). Each data point represents the mean from triplicate determinations, and the error bar represents the standard error ACC, 1-aminocyclopropane-1-carboxylate. **(B)** Absorbance of bacterial isolates at different concentrations of ACC.

**FIGURE 8:**
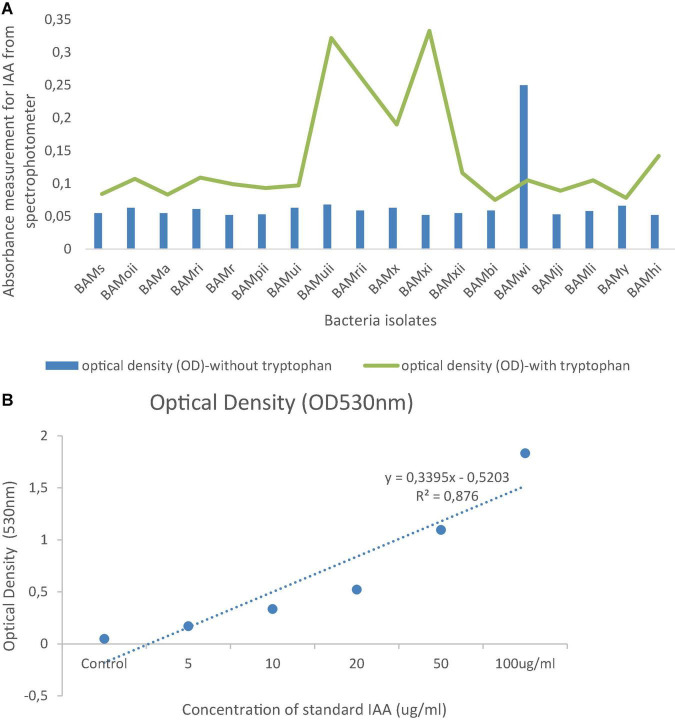
**(A)** Spectrophotometric measurement of absorbance of IAA in isolates in the presence or absence of tryptophan at optical density of 530 nm. **(B)** A standard graph of IAA at optical density of 530 nm.

From the PGPR tests, 41.87% (18) showed positive actions in two or more of the PGP tests. Out of which, 4.65% of the isolates were positive for HCN production, all were positive for NH_3_ production and ACC with absorbance at 570 nm, and a standard curve was drawn (Figures 7A,B). Isolates that were positive for IAA production were 16.28%. Absorbance value was recorded for all organisms (Figure 8A) and 27.91% solubilized phosphate (Table 2). The standard curve for IAA was also plotted (Figure 8B). This was used to calculate the quantity of IAA produced by each isolate (see Supplementary Table 1). Of all the isolates, 27.91% showed positive activity for catalase, oxidase, and protease production, but the isolates that were positive in at least two of the PGP tests were all positive in at least one of the biochemical tests, while 13.95% of the isolates that were not positive to at least two of the PGP tests were positive to all the three biochemical tests (Table 2). While out of the 18 isolates used in this study, 27.77% were positive to catalase, oxidase, and protease production.

**TABLE 2:** Plant growth-promoting activities and biochemical activities of rhizobacteria isolates from the abovementioned soil samples.

Isolates	IAA	NH_3_ production	HCN	P-solubilization	Catalase	Oxidase	Protease
BAMxi	+	++	–	++	+	+	–
BAMyi	–	+	–	++	++	–	–
BAMpii	+	+	–	–	+	+	–
BAMwi	+	+	–	–	+	+	–
BAMri	+	+	–	–	+	–	–
BAMoii	+	+	–	–	+	+	–
BAMxii	+	++	–	++	+	+	–
BAMuii	+	+	–	–	++	–	–
BAMr	–	+	–	+	++	+	+
BAMs	–	+	–	+	++	+	+
BAMhi	–	+	+	+	+	–	–
BAMli	–	++	–	+	++	+	+
BAMx	–	+	–	+	–	+	+
BAMa	–	+	–	++	+	–	–
BAMbi	–	+	–	++	+	+	+
BAMji	–	++	–	+	+	+	+
BAMui	–	+	+	–	+	–	–
BAMrii	–	+	–	+	+	–	–

*BAM means isolates are from Bambara groundnut rhizosphere, while the letters represent the different isolates. +, means positive; –, means negative; ++, means very positive.*

### Biocontrol Activities

Biocontrol activities were carried out in this study to test for the antifungal and antibacterial potentials of the bacterial isolates.

#### Antifungal Activities

**FIGURE 9:**
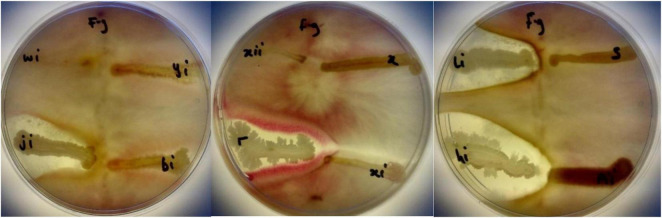
Antifungal activities of BAMji, BAMr, BAMli, and BAMhi against *F. graminearum* (*Source:* Ajilogba and Babalola, 2019).

Isolated bacteria were tested against *Fusarium graminearum* (written as f.g on the plate). BAMji, BAMr, BAMli, and BAMhi (9.3%) (*B. cereus*, *B. amyloliquefaciens*, *B. thuringiensis, Bacillus* sp.) showed antifungal potential against *F. graminearum* (Figure 9). The zone of inhibition reveals that *Bacillus* sp. inhibited the growth of *F. graminearum* the most (Table 3).

**TABLE 3:** A percentage area of inhibition for selected PGPR agents.

	Antifungal		
Rhizobacteria isolates	Radius of zone of inhibition (mm)	Area (mm^2^)	% area of inhibition
*Bacillus* sp (hi)	20	1256.6	15.9
*B. amyloliquefaciens* (r)	10	314.1	4
*B. thuringiensis* (li)	15	706.8	8.9
*B. cereus*(ji)	18	1017.8	12
*Fusarium graminearum*	50	7853.9	

The total inhibition area (πr^2^) = a. The total area of Petri dish (r = 50 mm) = b. The% area of inhibition = a/b*100.

#### Antibacterial Activities

**FIGURE 10:**
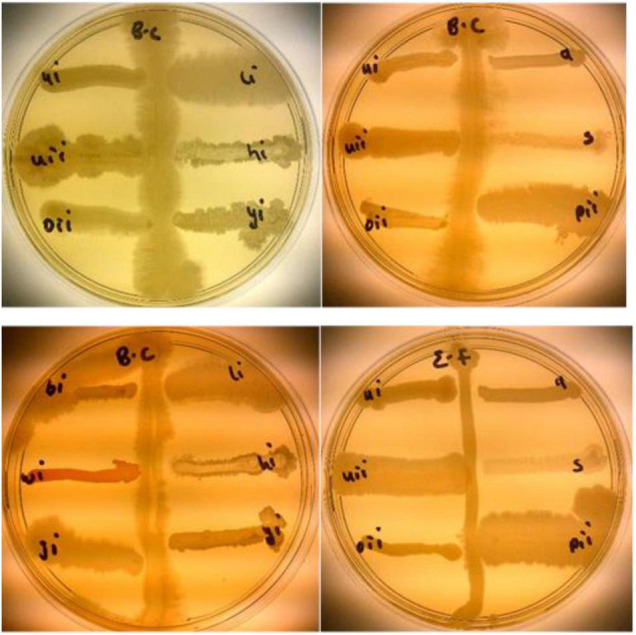
Antibacterial activities of isolates BAMui, BAMli, BAMoii, BAMyi, BAMhi, and BAMpii against *B. cereus* and *E. faecalis.*

Rhizobacteria from this study were tested against *B. cereus* (written as B.C on the plates) and *E. faecalis* (*written as* E.F *on the plates*). BAMui, BAMli, BAMoii, BAMyi, BAMhi, and BAMpii (16.2%) had antagonistic effects against *B. cereus* and *E. faecalis* as seen in the pattern formed on the streaked pathogen (Figure 10).

#### Characterization of Metabolites From Antifungal and Antibacterial Activities

**FIGURE 11:**
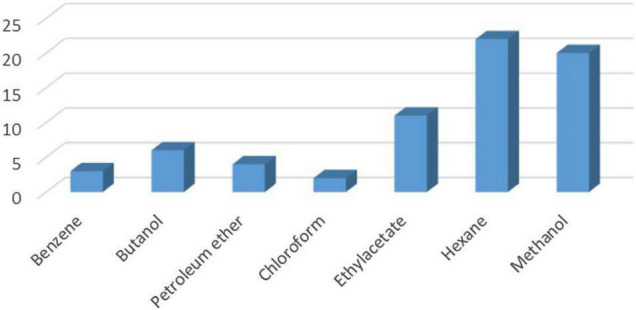
Total number of metabolites detected by GC–MS in *B. thuringiensis, B. amyloliquefaciens*, and *Bacillus* sp. using seven different extraction solvents (*Source:* Ajilogba and Babalola, 2019).

Characterization of the metabolites from *B. thuringiensis*, *B. amyloliquefaciens*, and *Bacillus* sp. (Figure 11) has already been reported in Ajilogba and Babalola (2019).

### Phylogenetic Analysis and Diversity

**FIGURE 12:**
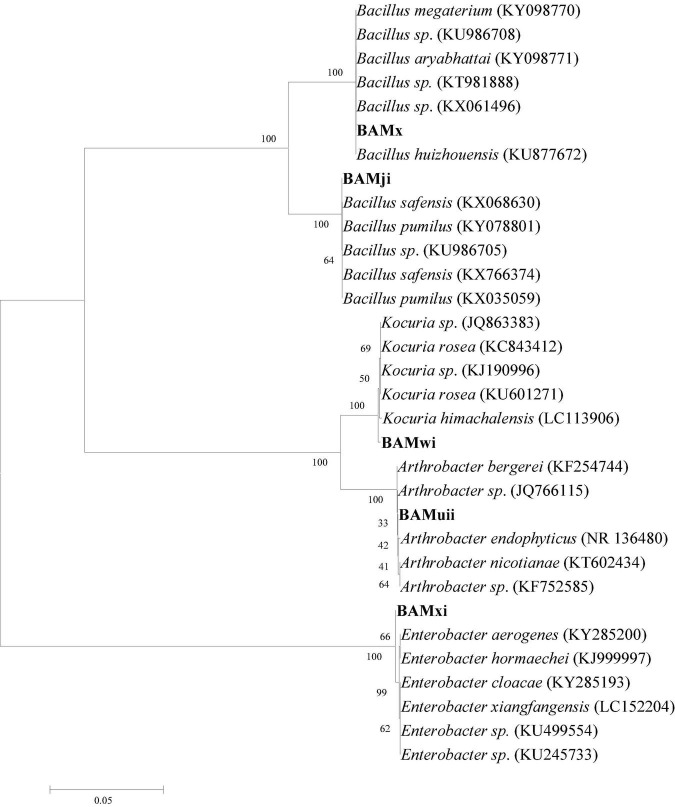
The phylogenetic tree based on 16S rRNA sequences using the neighbor-joining method for bacterial isolates and their closely related type strains.

The isolates were subjected to phylogenetic analysis. The 16S rDNA sequences of the bacterial isolates were aligned with reference nucleotide sequences obtained from the GenBank. The phylogenetic position of the bacterial isolates was evaluated by constructing a phylogenetic tree, using the neighbor-joining method (Figure 12). This method placed the bacterial isolates in different clades, encompassing members of their genera; this was supported with bootstrap values. The bootstrap values based on 1,000 replications were listed as percentages at the branching points.

### Molecular Identification of Selected Isolates

The 16S rDNA gene sequence of the selected isolates was obtained by BLASTn search; however, 27 strains of combination of the phylum firmicutes, actinobacteria, and proteobacteria were selected based on high identity (%), with good E value. The results in Table 3 show that query sequences were best pairwise aligned with the 16S rDNA gene sequence of other firmicutes, proteobacteria, and actinobacteria with sequence similarity and identity ranged between 96 and 99%, with *E*-value of 0.

### Supporting Data

The 16S rDNA gene sequences determined for the bacterial isolates in this study were deposited into the GenBank database and assigned accession numbers (Table 4).

**TABLE 4:** Results of 16S rDNA gene sequence similarities of rhizobacteria isolates and GenBank accession numbers using BLASTn algorithm isolate code.

Isolate code	Sequence length (bp)	Closest related strain in database	Accession number	Similarity (%)	*E*-value
BAMji	1424	*B. cereus*	KX588094	99	0
BAMr	1058	*Bacillus* sp.	KX588095	98	0
BAMui	1405	*Bacillus* sp.	KX588096	97	0
BAMx	1417	*B. megaterium*	KX588099	99	0
BAMuii	1353	*Glutamicibacter (Arthrobacter) bergerei*	KX588097	98	0
BAMwi	1335	*Kocuria rosea*	KX588098	99	0
BAMxi	1377	*Enterobacter hormaechei*	KX588100	99	0
BAMbi	1403	*B. safensis*	KX809651	99	0
BAMhi	1443	*B. amyloliquefaciens*	KX809652	99	0
BAMli	1388	*B. thuringiensis*	KX809653	99	0
BAMpii	1414	*M. hydrocarbonoxydans*	KX809654	99	0
BAMrii	1416	*M. testaceum*	KX809655	99	0
BAMxii	1389	*Acinetobacter parvus*	KX809656	98	0

### Growth Parameters Per Treatment

Mean of the growth parameter measured per plant is given on Table 4. This is a summary of the measurement taken per treatment on the field. The yield per plant of bambara groundnut on the field varied per treatment and compared with control as follows: *Bacillus sp, B. amyloliquefaciens, B thuringiensis*, and control produced average of 41, 36, 25, and 20 seeds, respectively. Treatment with *Bacillus sp* yielded more than double seeds compared with the control.

## Discussion

The plants’ rhizospheres, especially those of legumes, have been observed to be a microbiome for diverse PGPR. Bambara groundnut as a legminous plant is not only good for food but its rhizosphere is also very important in promoting plant growth and increasing food production. The rhizosphere is rich in total nitrogen, nitrate, and CEC. In this study, the nitrate available (Figure 3) in the soil falls in the same range with the soil nitrate analyzed at different growing seasons from Dobra Voda and Chvalina farms during spring and autumn, ranging between 4 and 14 ppm N, although some of the values from this study are higher (Vaněk et al., 2003). These higher values of nitrate can be as a result of bambara groundnut’s ability to fix nitrogen through symbiosis with the bacteria in the root nodules. According to Vaněk et al. (2003), it can also be as a result of the activities of leguminous crops in which case bambara groundnut is one. The CEC of soil samples in this study (between 15.19 and 21.64 meq/100 g) falls within the range of CEC of organically and conventionally managed apple orchard, ranging from 19.23 to 20.28 cmol/kg (Gasparatos et al., 2011), even though no fertilizer or organic manure was applied to bambara groundnut in this study.

Normally, leguminous plants are sensitive to pH, and this is important to their nitrogen-fixing ability because, at low pH, the soil is acidic and inhibits the activities of nitrogen-fixing bacteria, and nitrogen is not released for plant growth (Ferreira et al., 2016). Bambara groundnut grows well on acidic soils (Khan, 2021), which agrees with the result from this study. It was observed that there was an increase in alfalfa yield when there was no additional nitrogen, and the pH was high, which is in contrast to the observation in this study. Although the pH of the soils throughout the season was acidic as they ranged from 2.3 to 3.49, nitrogen was available in the form of nitrate as mentioned earlier, and bambara groundnut was enhanced in growth. Out of 12 isolates from the root nodule of bambara groundnut isolated from Cameroonian soil, 8 of them were able to grow at pH 3.5 in the soil that was acidic. This also reveals the potential of bambara groundnut to grow under very harsh condition (Laurette et al., 2015). Soil pH and redox potential (Eh) are negatively correlated, as when one increases, the other decreases. The Eh in this study ranged from 170.33 to 221.66 mV, which falls into the category of moderately reduced soils, having Eh between +100 and +400 mV and close to cultivated soils with an Eh range of between +300 and +500 mV (Husson, 2013). The Eh and pH of the soil help to determine the type of metabolism evident in the bacterial community of the soil and, invariably, the biological activities of the soil. Growth and development of soil bacteria, and their metabolic, enzymatic, and microbial activities are directly or indirectly affected by the Eh and/or the pH (Husson, 2013; Qu et al., 2020).

Soil organic matter in the soil is very vital and important as it can produce as much carbon found in the combination of that of the atmosphere as carbon dioxide and the biomass of plants (European Union, 2011; Princeton University Engineering School, 2021). In this study, the soil organic matter increased till 16 WAP and decreased at harvest; this could be as a result of heavy metabolic processes involving pod and seed formation at the 12 and 16 WAP. Assertion from this study points to the fact that between 4 WAP and 16 WAP, the soil of Bambara groundnut kept increasing and, at 16 WAP, experienced the highest level of fertility. At this point, other cereals can be cropped with Bambara groundnut to improve their growth.

Bacteria from the rhizosphere are important in auxin production and vitamin synthesis that encourage biofertilization (Ajilogba et al., 2022a). Bacterial isolates from this study were able to produce IAA and solubilize phosphate, which are important in biofertilization to increase crop growth. Ammonia, HCN and siderophore production, and phosphate solubilization are also able to contribute to biocontrol potentials of rhizobacteria (Laslo et al., 2012; Ajilogba and Babalola, 2013; Ajilogba et al., 2013, 2022a; Chukwuneme et al., 2020; dos Santos et al., 2020; Aasfar et al., 2021; Kumar et al., 2021). Soil phosphorous is an important macronutrient needed for plants to grow; hence, deficiency of phosphorus in soil is a major challenge in agricultural food/crop production.

Phosphate solubilization is a complex phenomenon, and it helps to discriminatively screen the bacteria that are able to break down tricalcium phosphate (TCP) and thereby release inorganic phosphate. According to Laslo et al. (2012), 63.8% of bacteria isolates from different rhizospheres of monocotyledonous plants solubilized phosphate as against 27.91% from this study. It was observed that none of the isolates from fields-growing chickpea from West of Allahabad Agricultural Institute, India produced HCN (Joseph et al., 2012), while, in this study, 4.65% produced HCN. This result is not comparable to a report by Agbodjato et al. (2015), which revealed that 100% of isolates from maize rhizosphere produced HCN.

Isolates BAMji, BAMhi, BAMli, and BAMr identified as *B. cereus*, *B. amyloliquefaciens*, *B. thuringiensis, Bacillus sp*, respectively, from this study were able to antagonize the growth of *F. graminearum*, which is an agricultural challenge to barley, wheat, and maize in South Africa (Boutigny et al., 2014; van der Lee et al., 2015). It was observed that *B. amyloliquefaciens* produced HCN in addition to ammonia and solubilizing phosphate, while *B. cereus*, *B. thuringiensis*, and *Bacillus* sp. produced ammonia and solubilized phosphate. Isolates BAMui, BAMoii, BAMyi, BAMpii, *B. amyloliquefaciens*, and *B. thuringiensis.* were able to antagonize the growth of *B. cereus* and *E. faecalis*. Three of the antifungal isolates also displayed antibacterial activities, which show that some rhizobacteria are both antifungal and antibacterial agents, while the rest are just purely antibacterial. The ability of these isolates to be able to inhibit and/or suppress the growth of both fungi and bacteria implies the richness of the bambara groundnut rhizosphere and its ability to resist diseases and pests.

The 16S rDNA gene sequence analysis was used to identify selected bacterial isolates in this study. The comparison of the bacterial isolate sequences revealed 96–99% identification similarities with the 16S rDNA gene sequence of the genus *Bacillus, Enterobacter, Arthrobacter*, and *Kocuria.*

### Relationship Between Bambara Groundnut Physical and Chemical Analyses, pH, Eh, Plant Growth-Promoting Rhizobacteria, and Biocontrol

Bambara groundnut physical and chemical analyses in this study reveal that the soil is an acidic soil, and the growth of Bambara groundnut in the soil makes it more acidic and that it can thrive in acidic soil. It has been observed that redox reaction is very important to the biocontrol activity of plant pathogen by rhizobacteria (Husson, 2013). This they do by generating reactive oxygen species (ROS) in the plant, and this serves as an antagonistic response of the plant to the pathogen and indirectly stresses the pathogen (Qi et al., 2018; Mansoor et al., 2022). Hydrogen peroxide and other signals like salicyclic and glutathione have been observed to increase the resistance of plants to pathogens (Herrera-Vásquez et al., 2015; Romero-Puertas et al., 2021). The isolates in this study were also able to release hydrogen peroxide in the catalase reaction, which might have also enhanced their ability to resist pathogen growth.

### Two-Year Field Trial of Identified Rhizobacteria

The impact of PGPR on plants in the same environment from where they were isolated is important in biofertilization. This is because the natural environment of the rhizobacteria helps them to establish in such an environment and enable their quick PGPR activities. When transferred to a different environment, the rhizobacteria require time to establish after competition with other resident bacteria. Rhizobacteria *B. amyloliquefaciens*, *B. thuringiensis*, and *Bacillus* sp. were able to stabilize in the soil and affect plant growth within the expected growth stage of bambara groundnut.

In this study, it was observed that the vegetative part increased significantly for stems, leaves, roots, and shoots compared to the control (Table 5). The number of stems of bambara groundnut recorded in this study ranged from 10 in the control at 4 WAP to 130 in harvest, with treatment with *Bacillus sp.* This is very high and comparable with those reported by other authors, which range from average of 23.97–28.87 recorded by Onwubiko et al. (2011) and number of branches/nodes/stems, ranging from 10 to 14 in the study by Effa et al. (2016).

**TABLE 5:** Mean measurement characteristics of the treatment per plant.

Treatment		Measurement characteristics							
		Number of stems	Number of leaves	Length of stem	Length of leaves	Breadth of leaves	Number of seeds	Length of shoots	Length of roots
WEEK 4	*B. thuringiensis*	12	54	8.1	1.5	0.9	1	1.5	9.5
	*B. amyloliquefaciens*	18	60	8	1.8	0.8	1	2	10
	*Bacillus sp*	18	78	8.4	1.9	1	2	2.2	10.1
	control	10	50	6	1	0.5	0.8	1	7
WEEK 8	*B. thuringiensis*	19	68	8.5	3.3	0.9	5	2	10.3
	*B. amyloliquefaciens*	20 ± 13.56	75 ± 40.94	8.9 ± 4.40	3.2 ± 0.59	1 ± 0.26	4 ± 4.23	2.9 ± 1.53	10.5 ± 2.65
	*Bacillus sp*	39 ± 42.59	119 ± 127.63	9.2 ± 13.68	4.7 ± 0.75	1.2 ± 0.96	6 ± 14.54	3 ± 9.02	13.8 ± 10.42
	control	15	60	7	3	0.6	3	1.5	8
WEEK 12	*B. thuringiensis*	51	117	10.5	4.8	1	11	2.9	13.1
	*B. amyloliquefaciens*	55 ± 12.42	135 ± 37.96	10.1 ± 3.18	4.3 ± 0.75	1.2 ± 0.28	10 ± 3.41	3.6 ± 0.81	15.2 ± 2.14
	*Bacillus sp*	69 ± 12.56	177 ± 37.69	12.9 ± 3.55	5.5 ± 0.79	1.4 ± 0.31	14 ± 2.41	4.1 ± 0.97	16.4 ± 1.93
	control	45	100	8	3.5	0.8	8	2	10
WEEK 16	*B. thuringiensis*	82 ± 6.68	228 ± 20.03	11.6 ± 1.16	5.3 ± 0.67	1.4 ± 0.58	16 ± 3.33	3.5 ± 0.75	21.5 ± 2.72
	*B. amyloliquefaciens*	91 ± 12.99	246 ± 38.82	12.7 ± 5.01	6.6 ± 0.64	1.8 ± 0.36	19 ± 3.47	4.4 ± 0.31	20.8 ± 2.20
	*Bacillus sp*	101	294	14.3	6.8	1.7	25	5	22.4
	control	70	200	10	4	1	12	3	15
HARVEST	*B. thuringiensis*	93 ± 9.91	276 ± 56.41	12.8 ± 2.63	6.8 ± 0.84	2.1 ± 0.35	25 ± 3.82	5.3 ± 0.51	25.8 ± 3.36
	*B. amyloliquefaciens*	118 ± 19.65	353 ± 58.95	13.9 ± 5.31	7.5 ± 0.85	2.5 ± 0.30	36 ± 11.44	5.7 ± 0.48	27.2 ± 5.58
	*Bacillus sp*	130 ± 35.48	421 ± 106.45	16 ± 7.56	8 ± 0.78	3 ± 0.37	41 ± 29.40	6.1 ± 5.28	30.5 ± 8.49
	control	80	260	10	5	2	20	5	20

The length of shoots in this study ranged between 1 cm in control and 6.1 cm at harvest, with the *Bacillus* sp. treatment. The length of shoots in this study falls within the range of 1-cm to 20-cm long, as recommended by Murevanhema and Jideani (2013). Also, studies by Chibarabada et al. (2015) were observed with a range between 11.68 and 17.8 cm; the difference might be as a result of different genotypes, varieties of landraces or cultivars planted, and/or the condition under which they were grown. The root length ranged from 7 cm at 4 WAP in control to 30.5 cm at harvest in the treatment BAMr compared to the range between 4.36 cm and 6.31 cm also observed by Chibarabada et al. (2015).

In this study, the mean number of leaves per plant ranged from 50 leaves in the control at 4 WAP to 421 leaves in treatment with *Bacillus* sp. at harvest, while Onuh and Christo (2011) recorded that the mean number of leaves ranged from 9 in the control at 28 days after planting (DAP) to 14.1 at 14 DAP in the treatment with 500 ml of water. The number of leaves in this study far exceeds what has been recorded as number of leaves in bambara groundnut.

The number of seeds in this study ranged from 0.8 in the control at 0 WAP to 41 seeds in treatment with *Bacillus sp.* at harvest. It was also observed that, at 4 and 8 WAP, some of the plants had started producing pods and seeds, which were also reported by Swanevelder (1998), that flowering and seeding in bambara groundnut can start as early as 30 DAP. There was an increase in seed yield in contrast with the report by Onuh and Christo (2011) whose mean seed yield ranged between 2.3 and 3.3 seeds. This increase can be linked to the *Bacillus* isolates treatment of the seeds as was recorded.

The results obtained from application of the biofertilized seeds are significantly different from the control. This shows that biofertilization improved the soil composition, so that minerals were easily made available to plants and then plant growth and crop yield were enhanced. The ability of the *Bacillus* isolates to increase bambara groundnut growth could be as a result of the PGP potentials of the isolates (Laili and Antonius, 2017, Ajilogba et al., 2022a,b; Ajilogba and Babalola, 2019). They were able to solubilize phosphate and produce indole acetic acid, ammonia, and hydrogen cyanide. These potentials have been harnessed by different bacterial isolates to improve growth.

Rhizobacteria isolates were characterized using 16Sr RNA. The molecular relationship of the isolates shows that 57.14% are from the phylum firmicutes (genus *Bacilli)*, while 28.57% are from phylum actinobacteria, and 14.28% are from the phylum proteobacteria. The actinobacteria and firmicutes are closer together as they are both gram positive and contain high guanine and cytosine content in their DNA, while proteobacteria are gram-negative bacteria. In this study, there were more firmicutes compared to actinobacteria and proteobacteria. This is in agreement with the community structure of the rhizosphere of vascular plants from the Antarctica, having firmicutes as the most abundant phyla, while firmicutes, actinobacteria, and proteobacteria as the three most abundant phyla (Teixeira et al., 2010). These three groups of bacteria, firmicutes, actinobacteria, and proteobacteria, have been described as important plant growth-promoting groups of bacteria (Lagos et al., 2015). This ability to promote growth has also been proved in this study as the three *Bacillus* strains increased plant growth.

## Conclusion

The rhizosphere of Bambara groundnut is very rich in terms of biotic and abiotic components. This study revealed that the physical and chemical properties of soil at different growth stages are different, and they affected the number, types, and diversities of bacteria of Bambara groundnut rhizosphere. It is also observed from this study that PGP activities of rhizobacteria from Bambara groundnut’s rhizosphere are comparable to those of other legumes and crops. The 3 bacillus isolates used in field planting of Bambara groundnut in this study showed that they improved growth compared to the control. *Bacillus* sp. improved growth more than the other two *B. amyloliquefaciens* and *B. thuringiensis*. These isolates have been proved to have potential as biofertilizers under field conditions being adapted to all the environmental challenges. Hence, they can be used as a substitute for chemical fertilizers and biocontrol agents against fungal and bacterial pathogens.

## Data Availability Statement

The datasets presented in this study can be found in online repositories. The names of the repository/repositories and accession number(s) can be found below: CA and OB (July, 2016) GenBank accession numbers KX588093 – KX588101, (February, 2016) GenBank accession numbers KX809651–KX809656. Metagenomics data: BioSample database with Submission ID: SUB3344633, BioProject ID: PRJNA422360 and samples were given BioSample accession: SAMN08176610 NCBI.

## Author Contributions

CA carried out the experiment with support from PA and RA. CA wrote the manuscript with support from OB. OB supervised the project. All authors contributed to the article and approved the submitted version.

## Conflict of Interest

The authors declare that the research was conducted in the absence of any commercial or financial relationships that could be construed as a potential conflict of interest.

## Publisher’s Note

All claims expressed in this article are solely those of the authors and do not necessarily represent those of their affiliated organizations, or those of the publisher, the editors and the reviewers. Any product that may be evaluated in this article, or claim that may be made by its manufacturer, is not guaranteed or endorsed by the publisher.
