# Supplementary material for: Bambara Groundnut Rhizobacteria Antimicrobial and Biofertilization Potential
Source: Front Plant Sci. 2022 Jul 13;13:854937. doi: 10.3389/fpls.2022.854937 (PMC9326403; doi:10.3389/fpls.2022.854937)
Supplement: Supplementary file 1 [file Data_Sheet_1.docx]

**Table 1**: ANOVA for comparison of treatments and their effect on the number of stems

| *Dependent Variable: Number of Stem* | | | | | | | | | | |  |  |
| --- | --- | --- | --- | --- | --- | --- | --- | --- | --- | --- | --- | --- |
| Source | Type III Sum of Squares | | df | | Mean Square | | F | | Sig. | |  |  |
| Model | 91093.100^a^ | | 8 | | 11386.638 | | 196.068 | | .000 | |  |  |
| WEEK | 24472.700 | | 4 | | 6118.175 | | 105.350 | | .000 | |  |  |
| TREATMENT | 2095.600 | | 3 | | 698.533 | | 12.028 | | .001 | |  |  |
| Error | 696.900 | | 12 | | 58.075 | |  | |  | |  |  |
| Total | 91790.000 | | 20 | |  | |  | |  | |  |  |
| *Multiple Comparisons* | | | | | | | | | | | | |
| *Dependent Variable: Number of Stems* | | | | | | | | | | | | |
| *Tukey HSD* | | | | | | | | | | | | |
| (I) TREATMENT | | (J) TREATMENT | | Mean Difference (I-J) | | Std. Error | | Sig. | | 95% Confidence Interval | | |
|  |  |  |  |  |  |  |  |  |  | Lower Bound | | Upper Bound |
| *B. thuringiensis* | | *B. amyloliquefaciens* | | -9.00 | | 4.820 | | .292 | | -23.31 | | 5.31 |
|  |  | *Bacillus* sp | | -20.00^*^ | | 4.820 | | .006 | | -34.31 | | -5.69 |
|  |  | Control | | 7.40 | | 4.820 | | .448 | | -6.91 | | 21.71 |
| *B. amyloliquefaciens* | | *B. thuringiensis* | | 9.00 | | 4.820 | | .292 | | -5.31 | | 23.31 |
|  |  | *Bacillus* sp | | -11.00 | | 4.820 | | .157 | | -25.31 | | 3.31 |
|  |  | Control | | 16.40^*^ | | 4.820 | | .023 | | 2.09 | | 30.71 |
| *Bacillus* sp | | *B. thuringiensis* | | 20.00^*^ | | 4.820 | | .006 | | 5.69 | | 34.31 |
|  |  | *B. amyloliquefaciens* | | 11.00 | | 4.820 | | .157 | | -3.31 | | 25.31 |
|  |  | Control | | 27.40^*^ | | 4.820 | | .001 | | 13.09 | | 41.71 |
| Control | | *B. thuringiensis* | | -7.40 | | 4.820 | | .448 | | -21.71 | | 6.91 |
|  |  | *B. amyloliquefaciens* | | -16.40^*^ | | 4.820 | | .023 | | -30.71 | | -2.09 |
|  |  | *Bacillus* sp | | -27.40^*^ | | 4.820 | | .001 | | -41.71 | | -13.09 |
| *Based on observed means.*  *The error term is Mean Square (Error) = 58.075.* | | | | | | | | | | | | |
| **The mean difference is significant at the .05 level.* | | | | | | | | | | | | |

**Table 2**: ANOVA for comparison of treatments and their effect on the number of leaves

| *Dependent Variable: Number of Leaves* | | | | | | | | | | |  |  |
| --- | --- | --- | --- | --- | --- | --- | --- | --- | --- | --- | --- | --- |
| Source | Type III Sum of Squares | | df | | Mean Square | | F | | Sig. | |  |  |
| Model | 794027.400^a^ | | 8 | | 99253.425 | | 172.925 | | .000 | |  |  |
| WEEK | 205621.200 | | 4 | | 51405.300 | | 89.561 | | .000 | |  |  |
| TREATMENT | 20224.150 | | 3 | | 6741.383 | | 11.745 | | .001 | |  |  |
| Error | 6887.600 | | 12 | | 573.967 | |  | |  | |  |  |
| Total | 800915.000 | | 20 | |  | |  | |  | |  |  |
| *Multiple Comparisons*  *Dependent Variable: Number of Leaves* | | | | | | | | | | | | |
| *Tukey HSD* | | | | | | | | | | | | |
| (I) TREATMENT | | (J) TREATMENT | | Mean Difference (I-J) | | Std. Error | | Sig. | | 95% Confidence Interval | | |
|  |  |  |  |  |  |  |  |  |  | Lower Bound | | Upper Bound |
| *B. thuringiensis* | | *B. amyloliquefaciens* | | -25.20 | | 15.152 | | .383 | | -70.19 | | 19.79 |
|  |  | *Bacillus* sp | | -69.20^*^ | | 15.152 | | .003 | | -114.19 | | -24.21 |
|  |  | Control | | 14.60 | | 15.152 | | .772 | | -30.39 | | 59.59 |
| *B. amyloliquefaciens* | | *B. thuringiensis* | | 25.20 | | 15.152 | | .383 | | -19.79 | | 70.19 |
|  |  | *Bacillus* sp | | -44.00 | | 15.152 | | .056 | | -88.99 | | .99 |
|  |  | Control | | 39.80 | | 15.152 | | .090 | | -5.19 | | 84.79 |
| *Bacillus* sp | | *B. thuringiensis* | | 69.20^*^ | | 15.152 | | .003 | | 24.21 | | 114.19 |
|  |  | *B. amyloliquefaciens* | | 44.00 | | 15.152 | | .056 | | -.99 | | 88.99 |
|  |  | Control | | 83.80^*^ | | 15.152 | | .001 | | 38.81 | | 128.79 |
| Control | | *B. thuringiensis* | | -14.60 | | 15.152 | | .772 | | -59.59 | | 30.39 |
|  |  | *B. amyloliquefaciens* | | -39.80 | | 15.152 | | .090 | | -84.79 | | 5.19 |
|  |  | *Bacillus* sp | | -83.80^*^ | | 15.152 | | .001 | | -128.79 | | -38.81 |
| *Based on observed means.*  *The error term is Mean Square (Error) = 573.967.* | | | | | | | | | | | | |
| **The mean difference is significant at the .05 level.* | | | | | | | | | | | | |

**Table 3**: ANOVA for comparison of treatments and their effect on the length of stem

| *Dependent Variable: Length of Stem* | | | | | | | | | | |  |  |
| --- | --- | --- | --- | --- | --- | --- | --- | --- | --- | --- | --- | --- |
| Source | Type III Sum of Squares | | df | | Mean Square | | F | | Sig. | |  |  |
| Model | 2254.451^a^ | | 8 | | 281.806 | | 147.807 | | .000 | |  |  |
| WEEK | 89.797 | | 4 | | 22.449 | | 11.775 | | .000 | |  |  |
| TREATMENT | 24.274 | | 3 | | 8.091 | | 4.244 | | .029 | |  |  |
| Error | 22.879 | | 12 | | 1.907 | |  | |  | |  |  |
| Total | 2277.330 | | 20 | |  | |  | |  | |  |  |
| Multiple Comparisons | | | | | | | | | | | | |
| Dependent Variable: Length of Stem | | | | | | | | | | | | |
| Tukey HSD | | | | | | | | | | | | |
| (I) TREATMENT | | (J) TREATMENT | | Mean Difference (I-J) | | Std. Error | | Sig. | | 95% Confidence Interval | | |
|  |  |  |  |  |  |  |  |  |  | Lower Bound | | Upper Bound |
| *B. thuringiensis* | | *B. amyloliquefaciens* | | -2.040 | | .8733 | | .144 | | -4.633 | | .553 |
|  |  | *Bacillus* sp | | -2.000 | | .8733 | | .155 | | -4.593 | | .593 |
|  |  | Control | | .340 | | .8733 | | .979 | | -2.253 | | 2.933 |
| *B. amyloliquefaciens* | | *B. thuringiensis* | | 2.040 | | .8733 | | .144 | | -.553 | | 4.633 |
|  |  | *Bacillus* sp | | .040 | | .8733 | | 1.000 | | -2.553 | | 2.633 |
|  |  | Control | | 2.380 | | .8733 | | .076 | | -.213 | | 4.973 |
| *Bacillus* sp | | *B. thuringiensis* | | 2.000 | | .8733 | | .155 | | -.593 | | 4.593 |
|  |  | *B. amyloliquefaciens* | | -.040 | | .8733 | | 1.000 | | -2.633 | | 2.553 |
|  |  | Control | | 2.340 | | .8733 | | .082 | | -.253 | | 4.933 |
| Control | | *B. thuringiensis* | | -.340 | | .8733 | | .979 | | -2.933 | | 2.253 |
|  |  | *B. amyloliquefaciens* | | -2.380 | | .8733 | | .076 | | -4.973 | | .213 |
|  |  | *Bacillus* sp | | -2.340 | | .8733 | | .082 | | -4.933 | | .253 |
| Based on observed means.  The error term is Mean Square (Error) = 1.907. | | | | | | | | | | | | |

**Table 4**: ANOVA for comparison of treatments and their effect on the length of leaves

| *Dependent Variable: Length of Leaves* | | | | | | | | | | |  |  |
| --- | --- | --- | --- | --- | --- | --- | --- | --- | --- | --- | --- | --- |
| Source | Type III Sum of Squares | | df | | Mean Square | | F | | Sig. | |  |  |
| Model | 465.185^a^ | | 8 | | 58.148 | | 106.612 | | .000 | |  |  |
| WEEK | 65.455 | | 4 | | 16.364 | | 30.002 | | .000 | |  |  |
| TREATMENT | 8.117 | | 3 | | 2.706 | | 4.961 | | .018 | |  |  |
| Error | 6.545 | | 12 | | .545 | |  | |  | |  |  |
| Total | 471.730 | | 20 | |  | |  | |  | |  |  |
| *Multiple Comparisons*  *Dependent Variable: Length of Leaves* | | | | | | | | | | | | |
| *Tukey HSD* | | | | | | | | | | | | |
| (I) TREATMENT | | (J) TREATMENT | | Mean Difference (I-J) | | Std. Error | | Sig. | | 95% Confidence Interval | | |
|  |  |  |  |  |  |  |  |  |  | Lower Bound | | Upper Bound |
| *B. thuringiensis* | | *B. amyloliquefaciens* | | -.840 | | .4671 | | .321 | | -2.227 | | .547 |
|  |  | *Bacillus* sp | | -1.060 | | .4671 | | .160 | | -2.447 | | .327 |
|  |  | Control | | .520 | | .4671 | | .689 | | -.867 | | 1.907 |
| *B. amyloliquefaciens* | | *B. thuringiensis* | | .840 | | .4671 | | .321 | | -.547 | | 2.227 |
|  |  | *Bacillus* sp | | -.220 | | .4671 | | .964 | | -1.607 | | 1.167 |
|  |  | Control | | 1.360 | | .4671 | | .055 | | -.027 | | 2.747 |
| *Bacillus* sp | | *B. thuringiensis* | | 1.060 | | .4671 | | .160 | | -.327 | | 2.447 |
|  |  | *B. amyloliquefaciens* | | .220 | | .4671 | | .964 | | -1.167 | | 1.607 |
|  |  | Control | | 1.580^*^ | | .4671 | | .024 | | .193 | | 2.967 |
| Control | | *B. thuringiensis* | | -.520 | | .4671 | | .689 | | -1.907 | | .867 |
|  |  | *B. amyloliquefaciens* | | -1.360 | | .4671 | | .055 | | -2.747 | | .027 |
|  |  | *Bacillus* sp | | -1.580^*^ | | .4671 | | .024 | | -2.967 | | -.193 |
| *Based on observed means.*  *The error term is Mean Square (Error) = .545.* | | | | | | | | | | | | |
| **The mean difference is significant at the .05 level.* | | | | | | | | | | | | |

**Table 5**: ANOVA for comparison of treatments and their effect on the breadth of leaves

| *Dependent Variable: Breadth of Leaves* | | | | | | | | | | |  |  |
| --- | --- | --- | --- | --- | --- | --- | --- | --- | --- | --- | --- | --- |
| Source | Type III Sum of Squares | | df | | Mean Square | | F | | Sig. | |  |  |
| Model | 42.301^a^ | | 8 | | 5.288 | | 54.747 | | .000 | |  |  |
| WEEK | 7.645 | | 4 | | 1.911 | | 19.789 | | .000 | |  |  |
| TREATMENT | .856 | | 3 | | .285 | | 2.954 | | .075 | |  |  |
| Error | 1.159 | | 12 | | .097 | |  | |  | |  |  |
| Total | 43.460 | | 20 | |  | |  | |  | |  |  |
| *Multiple Comparisons*  *Dependent Variable: Breadth of Leaves* | | | | | | | | | | | | |
| *Tukey HSD* | | | | | | | | | | | | |
| (I) TREATMENT | | (J) TREATMENT | | Mean Difference (I-J) | | Std. Error | | Sig. | | 95% Confidence Interval | | |
|  |  |  |  |  |  |  |  |  |  | Lower Bound | | Upper Bound |
| *B. thuringiensis* | | *B. amyloliquefaciens* | | -.160 | | .1966 | | .847 | | -.744 | | .424 |
|  |  | *Bacillus* sp | | -.320 | | .1966 | | .400 | | -.904 | | .264 |
|  |  | Control | | .240 | | .1966 | | .626 | | -.344 | | .824 |
| *B. amyloliquefaciens* | | *B. thuringiensis* | | .160 | | .1966 | | .847 | | -.424 | | .744 |
|  |  | *Bacillus* sp | | -.160 | | .1966 | | .847 | | -.744 | | .424 |
|  |  | Control | | .400 | | .1966 | | .229 | | -.184 | | .984 |
| *Bacillus* sp | | *B. thuringiensis* | | .320 | | .1966 | | .400 | | -.264 | | .904 |
|  |  | *B. amyloliquefaciens* | | .160 | | .1966 | | .847 | | -.424 | | .744 |
|  |  | Control | | .560 | | .1966 | | .061 | | -.024 | | 1.144 |
| Control | | *B. thuringiensis* | | -.240 | | .1966 | | .626 | | -.824 | | .344 |
|  |  | *B. amyloliquefaciens* | | -.400 | | .1966 | | .229 | | -.984 | | .184 |
|  |  | *Bacillus* sp | | -.560 | | .1966 | | .061 | | -1.144 | | .024 |
| *Based on observed means.*  *The error term is Mean Square (Error) = .097.* | | | | | | | | | | | | |

**Table 6**: ANOVA for comparison of treatments and their effect on the number of seeds

| *Dependent Variable: Number of Seeds* | | | | | | | | | | |  |  |
| --- | --- | --- | --- | --- | --- | --- | --- | --- | --- | --- | --- | --- |
| Source | Type III Sum of Squares | | df | | Mean Square | | F | | Sig. | |  |  |
| Model | 5776.496^a^ | | 8 | | 722.062 | | 46.800 | | .000 | |  |  |
| WEEK | 2191.208 | | 4 | | 547.802 | | 35.505 | | .000 | |  |  |
| TREATMENT | 210.486 | | 3 | | 70.162 | | 4.548 | | .024 | |  |  |
| Error | 185.144 | | 12 | | 15.429 | |  | |  | |  |  |
| Total | 5961.640 | | 20 | |  | |  | |  | |  |  |
| *Multiple Comparisons*  *Dependent Variable: Number of Seeds* | | | | | | | | | | | | |
| *Tukey HSD* | | | | | | | | | | | | |
| (I) TREATMENT | | (J) TREATMENT | | Mean Difference (I-J) | | Std. Error | | Sig. | | 95% Confidence Interval | | |
|  |  |  |  |  |  |  |  |  |  | Lower Bound | | Upper Bound |
| *B. thuringiensis* | | *B. amyloliquefaciens* | | -2.40 | | 2.484 | | .771 | | -9.78 | | 4.98 |
|  |  | *Bacillus* sp | | -6.00 | | 2.484 | | .127 | | -13.38 | | 1.38 |
|  |  | Control | | 2.84 | | 2.484 | | .671 | | -4.54 | | 10.22 |
| *B. amyloliquefaciens* | | *B. thuringiensis* | | 2.40 | | 2.484 | | .771 | | -4.98 | | 9.78 |
|  |  | *Bacillus* sp | | -3.60 | | 2.484 | | .495 | | -10.98 | | 3.78 |
|  |  | Control | | 5.24 | | 2.484 | | .205 | | -2.14 | | 12.62 |
| *Bacillus* sp | | *B. thuringiensis* | | 6.00 | | 2.484 | | .127 | | -1.38 | | 13.38 |
|  |  | *B. amyloliquefaciens* | | 3.60 | | 2.484 | | .495 | | -3.78 | | 10.98 |
|  |  | Control | | 8.84^*^ | | 2.484 | | .018 | | 1.46 | | 16.22 |
| Control | | *B. thuringiensis* | | -2.84 | | 2.484 | | .671 | | -10.22 | | 4.54 |
|  |  | *B. amyloliquefaciens* | | -5.24 | | 2.484 | | .205 | | -12.62 | | 2.14 |
|  |  | *Bacillus* sp | | -8.84^*^ | | 2.484 | | .018 | | -16.22 | | -1.46 |
| *Based on observed means.*  *The error term is Mean Square (Error) = 15.429.* | | | | | | | | | | | | |
| **The mean difference is significant at the .05 level.* | | | | | | | | | | | | |

**Table 7**: ANOVA for comparison of treatments and their effect on the length of shoots

| *Dependent Variable: Length of Stems* | | | | | | | | | | |  |  |
| --- | --- | --- | --- | --- | --- | --- | --- | --- | --- | --- | --- | --- |
| Source | Type III Sum of Squares | | df | | Mean Square | | F | | Sig. | |  |  |
| Model | 265.233^a^ | | 8 | | 33.154 | | 362.670 | | .000 | |  |  |
| WEEK | 35.863 | | 4 | | 8.966 | | 98.076 | | .000 | |  |  |
| TREATMENT | 6.925 | | 3 | | 2.308 | | 25.253 | | .000 | |  |  |
| Error | 1.097 | | 12 | | .091 | |  | |  | |  |  |
| Total | 266.330 | | 20 | |  | |  | |  | |  |  |
| *Multiple Comparisons*  *Dependent Variable: Length of Shoots* | | | | | | | | | | | | |
| *Tukey HSD* | | | | | | | | | | | | |
| (I) TREATMENT | | (J) TREATMENT | | Mean Difference (I-J) | | Std. Error | | Sig. | | 95% Confidence Interval | | |
|  |  |  |  |  |  |  |  |  |  | Lower Bound | | Upper Bound |
| *B. thuringiensis* | | *B. amyloliquefaciens* | | -.820^*^ | | .1912 | | .005 | | -1.388 | | -.252 |
|  |  | *Bacillus* sp | | -1.020^*^ | | .1912 | | .001 | | -1.588 | | -.452 |
|  |  | Control | | .420 | | .1912 | | .179 | | -.148 | | .988 |
| *B. amyloliquefaciens* | | *B. thuringiensis* | | .820^*^ | | .1912 | | .005 | | .252 | | 1.388 |
|  |  | *Bacillus* sp | | -.200 | | .1912 | | .727 | | -.768 | | .368 |
|  |  | Control | | 1.240^*^ | | .1912 | | .000 | | .672 | | 1.808 |
| *Bacillus* sp | | *B. thuringiensis* | | 1.020^*^ | | .1912 | | .001 | | .452 | | 1.588 |
|  |  | *B. amyloliquefaciens* | | .200 | | .1912 | | .727 | | -.368 | | .768 |
|  |  | Control | | 1.440^*^ | | .1912 | | .000 | | .872 | | 2.008 |
| Control | | *B. thuringiensis* | | -.420 | | .1912 | | .179 | | -.988 | | .148 |
|  |  | *B. amyloliquefaciens* | | -1.240^*^ | | .1912 | | .000 | | -1.808 | | -.672 |
|  |  | *Bacillus* sp | | -1.440^*^ | | .1912 | | .000 | | -2.008 | | -.872 |
| *Based on observed means.*  *The error term is Mean Square (Error) = .091.* | | | | | | | | | | | | |
| **The mean difference is significant at the .05 level.* | | | | | | | | | | | | |

**Table 8**: ANOVA for comparison of treatments and their effect on the length of roots

| *Dependent Variable: Length of Roots* | | | | | | | | | | |  |  |
| --- | --- | --- | --- | --- | --- | --- | --- | --- | --- | --- | --- | --- |
| Source | Type III Sum of Squares | | df | | Mean Square | | F | | Sig. | |  |  |
| Model | 5829.589^a^ | | 8 | | 728.699 | | 219.537 | | .000 | |  |  |
| WEEK | 790.505 | | 4 | | 197.626 | | 59.539 | | .000 | |  |  |
| TREATMENT | 109.284 | | 3 | | 36.428 | | 10.975 | | .001 | |  |  |
| Error | 39.831 | | 12 | | 3.319 | |  | |  | |  |  |
| Total | 5869.420 | | 20 | |  | |  | |  | |  |  |
| *Multiple Comparisons* | | | | | | | | | | | | |
| *Dependent Variable: Length of Roots* | | | | | | | | | | | | |
| *Tukey HSD* | | | | | | | | | | | | |
| (I) TREATMENT | | (J) TREATMENT | | Mean Difference (I-J) | | Std. Error | | Sig. | | 95% Confidence Interval | | |
|  |  |  |  |  |  |  |  |  |  | Lower Bound | | Upper Bound |
| *B. thuringiensis* | | *B. amyloliquefaciens* | | -1.980 | | 1.1523 | | .357 | | -5.401 | | 1.441 |
|  |  | *Bacillus* sp | | -2.560 | | 1.1523 | | .172 | | -5.981 | | .861 |
|  |  | Control | | 3.420 | | 1.1523 | | .050 | | -.001 | | 6.841 |
| *B. amyloliquefaciens* | | *B. thuringiensis* | | 1.980 | | 1.1523 | | .357 | | -1.441 | | 5.401 |
|  |  | *Bacillus* sp | | -.580 | | 1.1523 | | .957 | | -4.001 | | 2.841 |
|  |  | Control | | 5.400^*^ | | 1.1523 | | .003 | | 1.979 | | 8.821 |
| *Bacillus* sp | | *B. thuringiensis* | | 2.560 | | 1.1523 | | .172 | | -.861 | | 5.981 |
|  |  | *B. amyloliquefaciens* | | .580 | | 1.1523 | | .957 | | -2.841 | | 4.001 |
|  |  | Control | | 5.980^*^ | | 1.1523 | | .001 | | 2.559 | | 9.401 |
| Control | | *B. thuringiensis* | | -3.420 | | 1.1523 | | .050 | | -6.841 | | .001 |
|  |  | *B. amyloliquefaciens* | | -5.400^*^ | | 1.1523 | | .003 | | -8.821 | | -1.979 |
|  |  | *Bacillus* sp | | -5.980^*^ | | 1.1523 | | .001 | | -9.401 | | -2.559 |
| *Based on observed means.*  *The error term is Mean Square (Error) = 3.319.* | | | | | | | | | | | | |
| **The mean difference is significant at the .05 level.* | | | | | | | | | | | | |
