# Supplementary material for: Bambara Groundnut Rhizobacteria Antimicrobial and Biofertilization Potential
Source: Front Plant Sci. 2022 Jul 13;13:854937. doi: 10.3389/fpls.2022.854937 (PMC9326403; doi:10.3389/fpls.2022.854937)
Supplement: Supplementary file 2 [file Table_1.docx]

**Table 1: IAA produced by each bacteria isolate calculated from the standard curve (**y=0.3395x**-**0.5203)

|  | **Absorbance at 530nm** | | **IAA quantified (ug/ml)** | |
| --- | --- | --- | --- | --- |
| **Bacteria isolates** | **Without tryptophan** | **With tryptophan** | **Without tryptophan** | **With tryptophan** |
| BAMs | 0.055 | 0.084 | 1.69 | 1.78 |
| BAMoii | 0.063 | 0.107 | 1.72 | 1.85 |
| BAMa | 0.055 | 0.083 | 1.69 | 1.78 |
| BAMri | 0.061 | 0.109 | 1.71 | 1.85 |
| BAMr | 0.052 | 0.099 | 1.69 | 1.82 |
| BAMpii | 0.053 | 0.093 | 1.69 | 1.82 |
| BAMui | 0.063 | 0.097 | 1.72 | 1.82 |
| BAMuii | 0.068 | 0.322 | 1.73 | 2.43 |
| BAMrii | 0.059 | 0.256 | 1.71 | 2.29 |
| BAMx | 0.063 | 0.19 | 1.72 | 2.09 |
| BAMxi | 0.052 | 0.333 | 1.69 | 2.51 |
| BAMxii | 0.055 | 0.116 | 1.69 | 1.87 |
| BAMbi | 0.059 | 0.075 | 1.71 | 1.75 |
| BAMwi | 0.25 | 0.105 | 2.27 | 1.84 |
| BAMji | 0.053 | 0.089 | 1.69 | 1.79 |
| BAMli | 0.058 | 0.105 | 1.7 | 1.84 |
| BAMy | 0.066 | 0.078 | 1.73 | 1.76 |
| BAMhi | 0.052 | 0.142 | 1.69 | 1.95 |
